# Post-endoscopic Retrograde Cholangiopancreatography Hemorrhagic Pancreatitis in a Young Female: A Case Report

**DOI:** 10.7759/cureus.60929

**Published:** 2024-05-23

**Authors:** Ibrahim Shanti, Malik Samardali, Zarna Bambhroliya, Leena Alhusari

**Affiliations:** 1 Internal Medicine, Marshall University Joan C. Edwards School of Medicine, Huntington, USA

**Keywords:** acute pancreatitis, abdominal pain, post-ercp pancreatitis (pep), cholelithiasis, hemorrhagic pancreatitis, endoscopy ercp

## Abstract

Hemorrhagic pancreatitis following endoscopic retrograde cholangiopancreatography (ERCP) is an adverse event that has received limited attention in medical studies. We describe a 28-year-old female who was admitted with symptoms of abdominal pain, nausea, and vomiting, along with tenderness in the right upper quadrant upon physical examination. CT abdomen revealed the presence of a gallstone obstructing the common bile duct. The patient underwent an ERCP procedure, which included a biliary sphincterotomy and the balloon-assisted removal of the obstructing stone. Unfortunately, the procedure was complicated with acute pancreatitis characterized by fluid accumulation in the abdomen, suggestive of hemorrhagic pancreatitis. There was a notable decrease in hemoglobin levels and hypotension, indicating the need for a higher level of care. Patients were managed conservatively with hydration and pain control. Follow-up in the clinic confirmed the resolution of symptoms and stabilization of the hemoglobin. Prompt recognition of post-ERCP hemorrhagic pancreatitis is crucial and warrants a high index of suspicion. Furthermore, the discussion explored the various risk factors and pathological events behind post-ERCP pancreatitis to understand the mechanisms of the disease. Various previously used intervention and prevention strategies were critically discussed for the awareness of future researchers and healthcare practitioners.

## Introduction

In modern medicine, the most common abdominal pathology is acute pancreatitis, accounting for 25% of all cases [1]. Acute pancreatitis is a life-threatening condition that presents severe abdominal discomfort commonly requiring hospitalization with an estimated mortality rate of 15-20% [1,2]. Hemorrhage pancreatitis is uncommon but has the potential to cause death due to the lack of timely diagnosis and treatment. The incidence rate of hemorrhagic pancreatitis is reported to be approximately 1-23% due to the rarity of this complication. However, this rare complication led to mortality in half of reported cases [3]. In the peripancreatic area, the hemorrhage further complicates and causes necrosis, ultimately leading to mortality. Generally, hemorrhage can be caused by the formation of pseudoaneurysms, mesenteric venous thrombosis, and erosion of vascular wall that conversely originates from esophagogastric dilation of veins and following surgical debridement [4]. The pathogenesis of hemorrhage pancreatitis is multifactorial. Gallstones are reported to be the most frequent risk factor of acute pancreatitis, other risk factors include alcohol consumption, hypertriglyceridemia, external trauma, post-endoscopic retrograde cholangiopancreatography (ERCP), and acute viral infection. Early pancreatitis hemorrhage arises within 24 hours of surgery due to perioperative coagulopathy and technical failures of inadequate hemostasis [5].

Post-ERCP pancreatitis (PEP) is defined as the condition of acute pancreatitis following post-ERCP and followed by clinical symptoms of increased levels of pancreatic enzymes [6]. The incidence of PEP is 10.7% among Asian patients as reported by 64 RCTs and 9% among European patients from 55 RCTs [7]. The post-ERCP is the most common serious adverse event also known as pancreatitis (PEP) with an estimated annual cost exceeding 150 million dollars in the United States [8]. Most other adverse events are of mild-to-moderate severity. Possible attributable independent risk factors for pancreatitis include anatomical variation, procedure-related variables, patient-specific traits, multiple cannulation attempts, brushing to procure pancreatic duct (PD) cytology specimens, and the development of pain during the procedure [9]. The risk of PEP increased gradually from 3.3% with less than 5 cannulation attempts to 14.9% when more than 20 attempts were made [10].

Interestingly, post-ERCP hemorrhagic pancreatitis is not well studied in the literature. In a recent case study, a similar case of a 28-year-old female who developed hemorrhagic pancreatitis following post-ERCP was reported. This case will help to understand clinical pathology, risk factors, and management of hemorrhagic pancreatitis.

## Case presentation

Our case is a 28-year-old female patient presented with six days of onset of nausea, vomiting, and abdominal pain. Her past medical history is significant for depression treated with a selective serotonin reuptake inhibitor.

She described experiencing a sharp, severe pain in her abdomen that radiated to her mid-back. She reported no fever, diarrhea, amenorrhea, or symptoms indicative of a urinary tract infection (UTI). She denied experiencing fever, diarrhea, amenorrhea, or UTI symptoms. Examination was evident for right upper quadrant discomfort, otherwise unremarkable exam. Laboratory results indicated a normal white blood cell count but showed evidence of elevated liver enzymes (Table 1). An ultrasound of the upper right quadrant revealed gallstones and a dilation of the common bile duct measuring 6.4 mm (about 0.25 in).

**Table 1:** Relevant laboratory results

Biochemical markers	Lab result	Reference range
White cell count	6	4.5-10 × 10^9/L
Hemoglobin	13	11-18 × 10^9/L
Platelets	330	150-440 × 10^9/L
Blood urea nitrogen (BUN)	15	5-18 × 10^9/L
Creatinine	0.8	0.7-1.4 × 10^9/L
International normalized ratio (INR)	1.1	1.3-1.7 gm/dL
Alanine aminotransferase (ALT)	749	4-36 U/L
Aspartate aminotransferase (AST)	839	10-36 units/L
Total bilirubin	2.4	0.1-1.2 mg/dL

**Figure 1:**
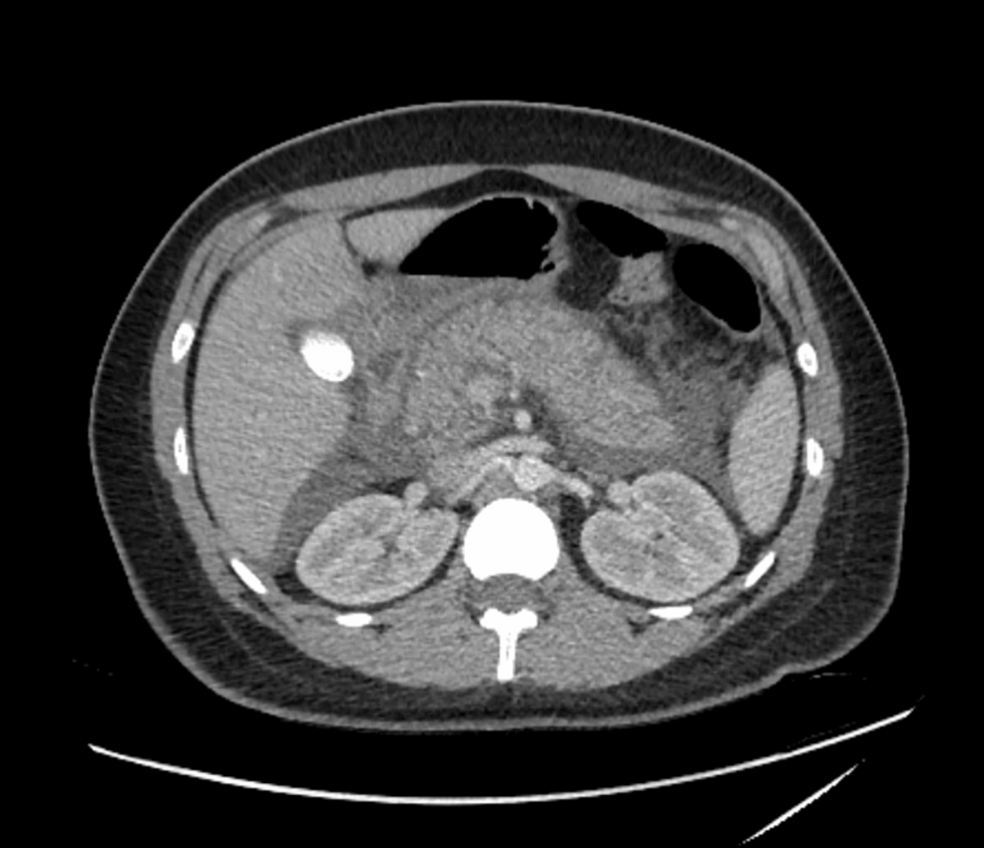
CT showing hemorrhagic pancreatitis

The patient was admitted to the hospital, and ERCP was performed smoothly which confirmed choledocholithiasis in CBD evident by filling defect compatible with stone and sludge on the cholangiogram. The patient underwent biliary sphincterotomy and balloon extraction of a 1 cm (about 0.39 in) stone, PD cannulation was not performed. There were no post-ERCP immediate complications. The patient started to complain of abdominal pain, nausea, and vomiting upon his return to the floor service. The examination was obvious for guarding and abdominal tenderness. Labs were significant for hemoglobin drop from 13 to 9 × 10^9/L (normal range: 11-18 × 10^9/L) and had elevated serum lipase of 600 U/L (normal range: 0 to 160 units per liter (U/L). Abdominal computed tomography (CT) (Figure 1) revealed signs of acute pancreatitis along with pneumobilia, which is most likely the result of the intervention.

In addition, complicated fluid was observed in the upper and lower right abdomen that had the density of blood in the images, which raised worries about possible bleeding and the possibility of hemorrhagic pancreatitis. The patient was transferred to the intensive care unit (ICU) for a higher level of care and was managed conservatively with intravenous fluids and pain medication. However, she did not require a transfusion of blood.

On the same admission, she underwent laparoscopy cholecystectomy and was discharged afterward. The patient was evaluated in the gastroenterology clinic two weeks later; she reported resolution of symptoms, no evidence of GI bleed, and hemoglobin levels were stable at 11 × 10^9/L.

## Discussion

ERCP is an extensively employed strategy for the diagnosis and treatment of disorders linked with the biliary system. Among complications of ERCP, PEP is regarded as common resulting in severe morbidity events and occasional death [3]. Other complications of ERCP are perforation, post-sphincterotomy hemorrhage, and progression of cholecystitis, reported rarely [6]. The average incidence rate of PEP is reported to be 5%, and approximately 35,000 cases of PEP are reported annually in the United States with an estimated cost of $199,500,000 [11]. In other reports, the annual cost spent on the treatment of PEP is $200 million [12]. As an ERCP-linked complication, hemorrhage pancreatitis is reported among 1-10% of total cases, but the risk of PEP is increasing as high as 20-25% among high-risk patients.

The PEP is associated with two types of factors; patient-related factors such as age and gender of the patient, sphincter of Oddi dysfunction (SOD), prior PEP, and pancreas divisum while procedure-related factors are PD injection, imbalanced homeostasis, and difficult cannulation [13]. It varies from minor concerns such as elevated amylase levels without clinical symptoms to more severe complications including cardiopulmonary depression, hypoxia, aspiration, intestinal perforation, hemorrhage, cholangitis, negative reactions to drugs, sepsis, acute pancreatitis, and in extreme cases, death [14].

Hemorrhagic pancreatitis following ERCP has not been extensively studied. Recently reported cases have attributed its etiology to procedural-related damage such as mechanical damage, heat-related injuries, and chemical harm to the pancreas [15]. Other symptoms such as six days of severe abdominal pain, and nausea followed by amylasemia showed the severity of the disorder and she needed hospitalization for 10 days with invasive treatment. Other pathophysiological mechanisms behind the occurrence of post-ERCP hemorrhage in recent cases are multiple and their contribution toward manifestations of disease are variable [16]. These can be pancreatic sphincter edema, mechanical injury of the pancreatic sphincter, and prolonged sphincter spasm in patients with sphincter hypertonia, as events of mechanical obstruction of pancreatic drainage and pancreatic bacterial contamination, production of hydrostatic hyper pressure in the duct and lack of experience for antiseptic measures during procedures among operators [17]. Since the extent to which any one of the mechanisms is involved is uncertain, numerous attempts have been made to determine each of the factors that contribute to the development of this issue. Prompt recognition of post-ERCP hemorrhagic pancreatitis is crucial, yet diagnosing it remains challenging due to overlapping symptoms with other post-ERCP complications and the need for sensitive imaging modalities to assess the extent of pancreatic hemorrhage accurately.

Numerous pharmacological and mechanical strategies are recommended for the prevention and treatment of PEP. In high-risk cases, the standard intervention is prophylactic pancreatic stents that reduce the severity of clinical manifestations by PEP. Studies proved that the use of prophylactic stenting of the PD reduces the risk of PEP [15]. Although the administration of rectal indomethacin has been promising in lowering the risk of PEP, there is a need for more randomized controlled trials (RCTs) to compare its effectiveness with that of PD stenting to confirm whether it can be considered an effective preventive measure on its own [16,18].

While pharmacological interventions have failed to treat the severity of PEP. Some intervention drugs such as somatostatin and gabexate showed improvement among PEP cases, but it is not commercially available globally and need continuous doses for improvements [13,14]. On the other hand, nonsteroidal anti-inflammatory drugs (NSAIDs) have proven to be effective and safe for the prevention of PEP as a low-cost and readily available therapeutic strategy. In inflammatory processes of acute pancreatitis, phospholipase A2 activity is dominant and found to be linked with arachidonic acid products, pro-inflammatory mediators, and platelet-activating factors, which are inhibited by NSAIDs, making it a potential treatment option [18]. Conclusively, prophylactic PD stent placement, rectal NSAIDs, and liberal administration of LR are considered potential therapeutic options for the treatment of PEP among high-risk patients [19]. Despite the advanced prevention strategies, the risk of hemorrhagic pancreatitis after ERCP persists. Therefore, identifying and addressing risk factors for bleeding, perforation, and cholangitis related to ERCP is crucial for reducing procedural risks and enhancing the safety of patients [20]. Further research to improve prevention techniques and pinpoint patients at high risk is essential in the primary prevention of post-ERCP complications.

Acute hemorrhagic pancreatitis can further progress to retroperitoneal hematoma. Although rare, a retroperitoneal hematoma can result in hemodynamic instability and symptoms due to compression, requiring prompt diagnosis and treatment. Our case highlights the importance of maintaining a high index of suspicion for such complications, as early recognition and management are crucial for optimal outcomes.

## Conclusions

In conclusion, our case highlights the rare but serious complication of hemorrhagic pancreatitis following ERCP, emphasizing the importance of prompt recognition and management. PEP is a common complication, but hemorrhagic pancreatitis is less studied and understood. This case underscores the need for a high index of suspicion for such complications, especially in high-risk patients. Preventive measures such as prophylactic pancreatic stents and rectal NSAIDs have shown promise in reducing the risk of PEP but may not eliminate the risk of hemorrhagic pancreatitis. Further research is needed to improve prevention strategies and identify patients at high risk. Overall, proactive risk assessment, timely intervention, and ongoing quality improvement initiatives are essential in minimizing the morbidity and mortality associated with ERCP-related complications.
